# Use of Yucca (*Yucca schidigera*) Extracts as Biostimulants to Promote Germination and Early Vigor and as Natural Fungicides

**DOI:** 10.3390/plants12020274

**Published:** 2023-01-06

**Authors:** Patricia Benito, Daniele Ligorio, Javier Bellón, Lynne Yenush, José M. Mulet

**Affiliations:** 1 Instituto de Biología Molecular y Celular de Plantas (IBMCP), Universitat Politècnica de València-Consejo Superior de Investigaciones Científicas, 46022 Valencia, Spain; 2 Caldic Ibérica, S. L. U. Llobateras 23–25, pol.ind. Santiga, Barberà del Vallés, 08210 Barcelona, Spain

**Keywords:** *Yucca schidigera*, *Brassica oleracea*, biostimulants, early vigor, natural fungicides, salt stress, drought stress, germination

## Abstract

Climate change is increasing drought and salinity in many cultivated areas, therefore threatening food production. There is a great demand for novel agricultural inputs able to maintain yield under the conditions imposed by the anthropogenic global warming. Biostimulants have been proposed as a useful tool to achieve this objective. We have investigated the biostimulant effect of different yucca (*Yucca schidigera*) extracts on plant growth at different stages of development under different abiotic stress conditions. The extracts were tested in the model plant *Arabidopsis thaliana*, and in three different crops; tomato (*Solanum lycopersicum* var *microtom*), broccoli (*Brassica oleracea* var. *italica*) and lettuce (*Lactuca sativa* var *romana*). We have found that the investigated extracts are able to promote germination and early vigor under drought/osmotic and salt stress induced either by sodium chloride or lithium chloride. This effect is particularly strong in *Arabidopsis thaliana* and in the Brassicaceae broccoli. We have also determined using antibiograms against the model yeast *Saccharomyces cerevisiae* that the evaluated extracts may be used also as a natural fungicide. The results in this report show that yucca extracts may be used to enhance early vigor in some crops and as a natural fungicide, providing a new and useful tool for farmers.

## 1. Introduction

Anthropogenic global warming is having an impact on climate variability, global temperatures, the biochemical state of the soil and sea level. The current measured temperature trend, which has been 0.6 ± 0.2 °C since 1900, appears to be increasing faster than expected. According to proposed models, the temperature is likely to continue increasing in a range between 1.4 and 5.8 °C during this century [1]. According to the European Copernicus Climate Change Service, the summer of 2022 was the warmest and driest in Europe and the second-warmest in the Northern Hemisphere since climatological records exist. A higher frequency of extreme events such as high-temperature, drought episodes and floods, can harm crops and reduce yields. Drought and salinity are among the most important environmental factors which will decrease agricultural production worldwide, and therefore are a major threat for food production. Both abiotic stress conditions can induce morphological, physiological, biochemical, and metabolic alterations through various mechanisms, eventually influencing plant growth, development and yield [2]. The ability of plants to respond to different environmental cues is associated with their plasticity, as well as the adaptability of plant traits to fluctuating water and salinity conditions, and the ability to withstand stress and resume development [3]. Continuous abiotic stress events can reduce the rate of germination and seedling growth [4]. The germination and early growth stages are the most vulnerable stages in the plant life cycle, as germination determines when and where seedling growth begins [5]. Several authors have described remarkable alterations in seed germination under saline stress in a wide variety of agricultural species, including some of the most widely cultivated crops such as maize [6], sorghum [7] and wheat [8]. This is because soil salinity can affect seed germination and development of young plants either by creating an osmotic potential external to the seed that prevents water uptake or through the toxic effects of Na^+^ and Cl^−^ ions on seed germination [9]. In addition, the excessive presence of salts in the soil can cause a nutritional imbalance resulting from the reduction of the absorption of some macro- or micronutrients [10].

Agriculture is undergoing a change in recent years to face the consequences of climate change, especially where unfavorable conditions drastically reduce yields and therefore the farmers’ income. The introduction of more efficient machinery, the use of new farming techniques and the transition to more sustainable production are giving rise to the use of new environmental-friendly products to encourage plant growth, such as agricultural biostimulants. Biostimulants have been defined as “substances and materials, with the exception of nutrients and pesticides, which, when applied to plants, seeds or growing substrates in specific formulations, have the capacity to modify physiological processes of plants in a way that provides potential benefits to growth, development and/or stress responses” [11]. Currently, there is growing interest in the biostimulant properties of plant extracts. This is because botanical extracts contain bioactive compounds that may improve flowering, plant growth, fruit set, crop productivity, and nutrient use efficiency, and may also improve tolerance against a wide variety of abiotic stressors [12]. In addition, the fact that they are natural products facilitates their use and commercialization under most regulations. Moreover, a large part of plant biodiversity remains unexplored for possible uses in agriculture. For these reasons, the analysis of botanical extracts as potential agents to help achieve the objective of sustainable agriculture is an increasingly active area of research. In addition, biostimulants have been proposed as a solution for mitigating the effects of climate change on crop performance and a powerful tool against the effects of abiotic stress [13,14].

Yucca is a genus of perennial succulent herbaceous plants, that grow in desert regions of the southwestern United States and northern Mexico [15]. *Yucca schidigera* is native to southwestern region of the United States and can also be found in north and central Mexico and in arid and semi-arid zones of southeastern United States. Originally, yucca plants were brought to Europe as ornamental plants. *Yucca gloriosa* was cultivated in English gardens about 1550 [16]. Yucca is still used as an ornamental plant, but starting in the XIX century, and its cultivation can be mechanized [17] and it is also cultivated for other purposes [18]. The leaf fiber is used to make textiles. Its flowers and fruits are edible, the seeds are milled to make flour and its roots are used to make soup. Some members of the family are cultivated for their medicinal properties [19,20,21]. The trunk is also edible and is used to produce yucca juice and yucca extract [15]. Byproducts of this process are bark, bagasse, and fine and coarse powders. The bark is rich in phenolic compounds and is a source of antioxidants [22]. Some of these byproducts have been valorized for other purposes, such as mushroom cultivation [23].

Yucca is used mainly in the form of extracts made by crushing stems of *Yucca schidigera* and other species of the genus Yucca. The main characteristic of this botanical extract is that it contains a high content of steroidal saponins and polyphenolic compounds [24]. The presence of these bioactive compounds in yucca extracts opens up a range of different applications, such as anti-inflammatories, antiarthritics [19], antimicrobials [25,26], surfactants, foaming agents in beverages, feed additives, atmospheric ammonia reducers [27,28], and crop growth stimulants [20]. Despite the commercial use of yucca extract as a plant biostimulant, there are few studies characterizing and moreover, quantifying its biostimulant effect on crops. This is the main reason that led us to undertake the present study. In this report, we have studied and quantified the effect of yucca extract on the model plant *Arabidopsis thaliana* and in crops such as broccoli, tomato and lettuce, in the presence of salt and osmotic stress. We have also evaluated its antifungal effect against the model yeast *Saccharomyces cerevisiae*.

## 2. Results

### 2.1. Evaluation of the Effect of Yucca Extracts on Germination and Early Development

**Figure 1:**
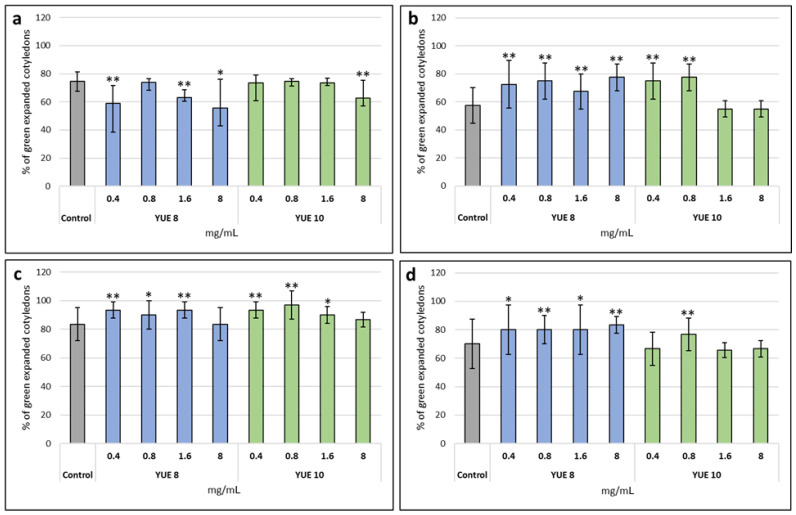
Determination of the effect of yucca extracts on the germination of (**a**) *A. thaliana* Col 0 WT, (**b**) broccoli, (**c**) tomato and (**d**) lettuce under normal conditions The *X* axis indicates the different concentrations (0.4, 0.8, 1.6 and 8 mg/mL) of the yucca extracts (control (gray); YUE 8 (blue) and YUE 10 (green)), while the *Y* axis represents the percentage of expanded green cotyledons. Statistical data from three experiments (n = 30 for each experiment) are presented. Bars represent the standard error * *p* < 0.05 by Student’s tests for increased germination compared to the control ** *p* < 0.01 by Student’s tests for increased germination compared to the control.

The objective of the present project was to determine the biological effect of two different yucca extracts. The difference among extracts was that YUE 8 had a low saponin content while YUE 10 had a high saponin content. First, we determined the effect of the extract on the germination and early development of different plants. We used a model plant (*Arabidopsis thaliana*) and three unrelated crops, the *Solanaceae tomato (*Solanum lycopersicum*), the *Brassicaceae* broccoli (*Brassica oleracea* var. Italica*) and the *Asteraceae* lettuce (*Lactuca sativa*). Yucca extracts had no growth-promoting effect in Arabidopsis (Figure 1a), in fact high concentrations showed an inhibitory effect. Low concentrations of yucca extract (0.4 or 0.8 mg/mL) improved early development in broccoli (Figure 1b) and to a lesser extent in tomato and lettuce (Figure 1c,d).

### 2.2. Effect of Yucca Extracts on Improving Abiotic Stress Resistance

**Figure 2:**
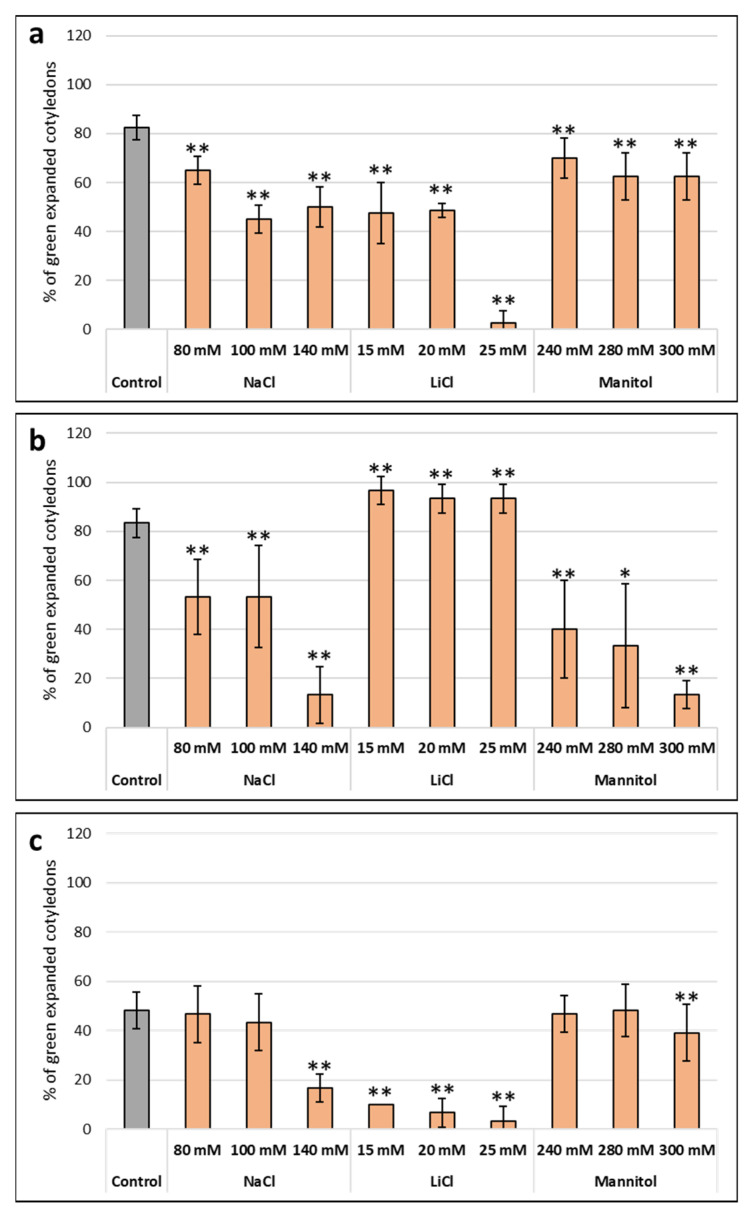
Evaluation of the effect of different concentrations of NaCl, LiCl and mannitol on the seed germination of (**a**) broccoli, (**b**) tomato and (**c**) lettuce. The *X*-axis indicates control without stress (gray) or the different concentrations of abiotic stressors (orange), while the *Y*-axis represents the percentage of expanded green cotyledons. The bars represent a standard error. * *p* < 0.05 by Student’s tests for increased germination compared to the control; ** *p* < 0.01 by Student’s tests for increased germination compared to the control.

Abiotic stress is a growing problem for agriculture. We wanted to check whether the yucca extracts were useful to alleviate the effects of drought or salt stress. For salt stress we used sodium and lithium. Plants may face the presence of sodium in the environment, so have developed systems to protect from sodium stress, while lithium is not present in most ecosystems and plants do not have specific mechanisms to protect from lithium stress. Concomitantly NaCl is toxic at higher concentrations than LiCl. At the concentrations used in this study NaCl has an osmotic effect in the medium, and therefore may induce water stress. Using lithium at lower concentrations we induce ionic toxicity in the plant and avoid the osmotic effect of sodium, so we can obtain information regarding whether the observed effect is due to ion toxicity within the cell or if it is due to the osmotic effect of sodium [29] In the absence of a toxic ion, we used mannitol to evaluate the osmotic effect. First, we determined the toxic concentration of each chemical for our non-model plants (Figure 2). We had determined the concentrations for Arabidopsis in a previous study [30]. 

**Figure 3:**
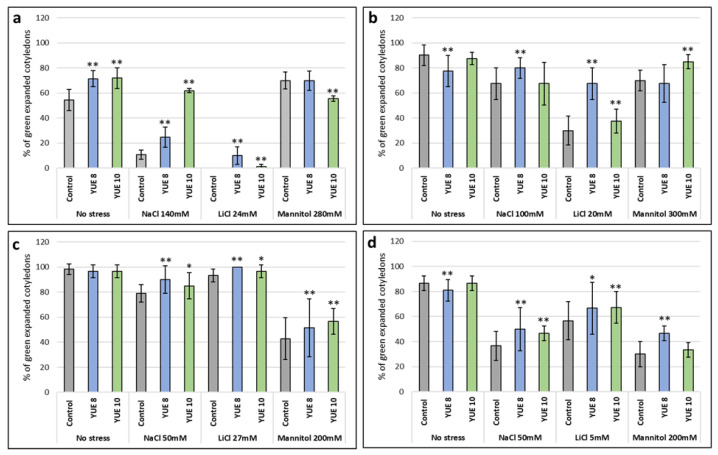
Effect of yucca extract on (**a**) *A. thaliana* Col 0 WT (**b**) broccoli, (**c**) tomato and (**d**) lettuce seed germination under control conditions (no stress) and saline (NaCl and LiCl) and osmotic (mannitol) stress. The optimal concentration of yucca extracts used in the different plant species tested was 0.8 mg/mL (*w*/*v*). The *X* axis indicates the different concentrations of abiotic stressors. Control without extract (gray); YUE 8 (blue) and YUE 10 (green), while the *Y* axis represents the percentage of expanded green cotyledons. The bars represent a standard error. * *p* < 0.05 by Student’s tests for increased germination compared to the control; ** *p* < 0.01 by Student’s tests for increased germination compared to the control.

**Figure 4:**
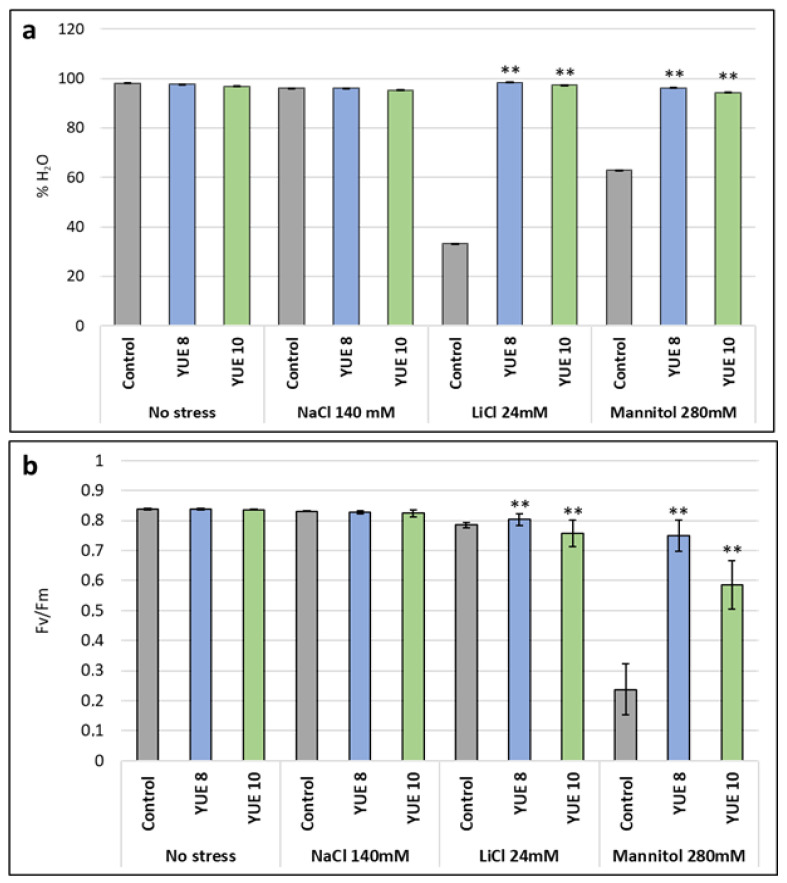
Effect of yucca extracts YUE 8 and YUE 10 on the physiological parameters of *A. thaliana* Col-0 WT plants under abiotic stress conditions. (**a**) Percentage of water content of the plants. (**b**) chlorophyll a yield index of the leaves. The *X*-axis indicates the different concentrations of abiotic stressors. Control without extract (gray); YUE 8 (blue) and YUE 10 (green), while the *Y*-axis represents the chlorophyll a yield index (Fv/Fm is presented as a ratio of variable fluorescence (Fv) over the maximum value of fluorescence (Fm)). The bars represent a standard error. ** indicates significant differences at *p* < 0.01 according to Student’s tests compared to the control.

According to the results of yucca extract concentrations determined in Figure 1, and salt and drought stress assayed in Figure 2, we selected appropriate concentrations to study whether the addition of yucca extract could influence the response to these abiotic stress conditions (see Figure 3 and Figure 4). The effect was very clear in the case of Arabidopsis and broccoli, (Figure 3a,b), in tomato and lettuce was not so strong but statistically significant in all conditions tested (Figure 3c,d). In general, YUE 8 was the extract that showed the greatest promotion of germination under saline stress.

### 2.3. Effect of Yucca Extracts on Physiological Parameters in Arabidopsis

We have determined that the most significant effect of yucca extracts was observed in the model plant *A. thaliana*. Specifically, under normal condition yucca extracts improved the average of green expanded cotyledons by a 71%, and under sodium chloride by a 100% (YUE 8) or 500% (YUE 10), Also YUE 8 promoted growth under lithium chloride, while under control conditions none of the seeds was able to germinate. We further characterized the effect by evaluating two different physiological parameters. Yucca extracts maintained the water content in Arabidopsis during lithium chloride and mannitol stress (Figure 4a). In respect to chlorophyll fluorescence, it slightly increased the levels with YUE 8 and slightly decreased with YUE 10 and increased the values to non-stressed levels during mannitol stress (Figure 4b). We could not observe any effect under sodium chloride stress. 

### 2.4. Characterization of the Fungicide Effect of Yucca Extracts

**Figure 5:**
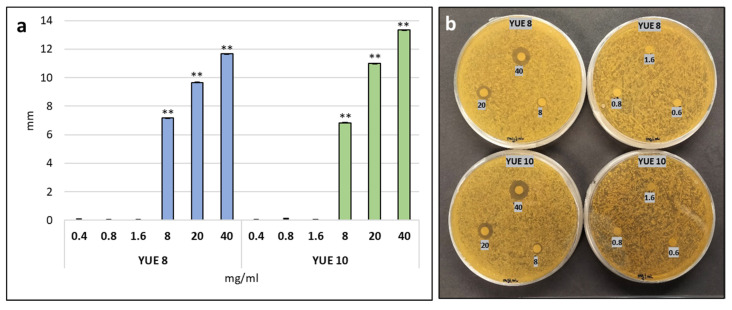
Antifungal effect of yucca extracts on yeast growth. (**a**) Inhibition halo (mm) of yeast BY4741 by YUE 8 (blue) and YUE 10 (green). (**b**) Halo formed around Whatman discs embedded with different concentrations (0.4, 0.8, 1.6, 8, 20 and 40 mg/mL) of YUE 8 and YUE 10. The bars represent the standard error. ** indicates significant differences at *p* < 0.01 according to the independent sample Student’s *t*-test.

We have found that yucca extracts have an effect on germination and early development. Yucca extracts are very rich in saponins, which are known to have fungicidal effects. Biostimulants are often used in combination with plant growth-promoting rhizobacteria (PGPR) or with mycorrhiza. Therefore, in order to complete the characterization, we investigated the behavior of our extracts with fungi. We used the model yeast *Saccharomyces cerevisiae* to measure the size of the halo formed by seriated dilutions of our extracts. (Figure 5). We could confirm and quantify the antifungal effect of yucca extracts. The concentrations tested can be found in Figure 5. Concentrations of yucca extracts above 8 mg/mL are deleterious for yeast. Specifically. for YUE 8 at 8 mg/mL, a growth inhibitory effect begins to be detected, with a halo of about 7 mm, becoming a halo of almost 12 mm in the case of 40 mg/mL. the highest concentration used. For YUE 10 at 8 mg/mL the halo is also about 7 mm, but at 40 mg/mL the halo is of almost 14 mm. 

## 3. Discussion

Biofertilizers and biostimulants are a potential solution to maintain agricultural yield despite demographic growth and anthropogenic global warming. They may stimulate plant growth, increase yield, reduce the abiotic stress impact, and decrease the fertilizer and pesticide dependence, which is a requirement for farmers due to the increasing price of energy [31]. 

It has been suggested that biostimulant investigation should focus upon finding proof of efficacy and safety and the determination of a broad mechanism of action, without a requirement for the determination of a specific mode of action or the molecular mechanisms [32]. We have developed a methodology to evaluate the efficacy of biostimulants using model organisms [33], and recently, we have further developed this methodology to identify synergies between different extracts [34]. We found that yucca extracts did not display any remarkable synergistic effect with other compounds, nor have any effect by themselves for promoting growth, but we found that during germination and early development, there was a remarkable effect on Arabidopsis seeds [34]. We have confirmed and quantified this initial observation and evaluated whether is a general effect or if it is limited to certain plant species. We have chosen three unrelated crops typical in Mediterranean agriculture (broccoli, tomato and lettuce), given that the Mediterranean basin is a semiarid area, and most climate models foresee that aridity will increase and will have a dramatic effect on forest [35] and arable land [36]. Early vigor is a desired trait that is limiting for many crops, especially cereals [37]. Increasing early vigor is a major objective in breeding programs [38], and there is also a major interest in enhancing early vigor under abiotic stress conditions with abiotic stress tolerance, because at this stage of development plants may be very sensitive to stress and this may lead to plant death [39]. Here, we present evidence of a plant extract which specifically can enhance early vigor in crop plants. Future studies will determine whether this extract is also effective in monocot plants, and therefore useful for cereal cultivation. It should be noted that as this product is a natural extract, it can be used in conventional or organic farming. Since the regulations for organic farming only allow products of natural origin [40], there is an urgent need for practices able to maintain yield in this kind of agriculture and novel inputs helping to this purpose. Biostimulants have been proposed as a solution [41]. In this report, we present evidence for a product suitable for organic farming that specifically may help farmers to increase early vigor. Moreover, we have shown that the yucca extract is toxic for yeast. The antifungal activity of yucca extracts has been associated with the presence of saponins, among other compounds [42]. Further studies will determine if these extracts are also toxic for plant pathogen fungi or, to other crops in order to determine the best cost-benefit relation and the optimal dose to get a positive effect with these extracts. 

Most of the emerging regulations are aimed at limiting the use of pesticides, including the farm-to-fork strategy of the European Union, thus limiting the availability of products useful for efficient plant protection. The number of fungicides available in organic farming is also very limited [43], and some of them are very contaminating and dangerous for the environment, such as copper, which is used mainly in grapevine, but also in other crops [44]. Here, we present initial evidence that yucca extracts could be an alternative for pest management with low environmental impact, as compared to copper, which is a persistent chemical, whereas saponins may be degraded in the environment by natural means [45]. This important characteristic lowers the possible environmental impact of yucca extract as its degradation products are 3–7 times less toxic than their parent products [46]. We have seen that, together with Arabidopsis, the most dramatic effect was observed in broccoli. This is in agreement with the fact that from the evolutionary point of view the closest cultivated relatives of Arabidopsis are the *Brassicaceae* family, which diverged about 17 million years [47]. This suggests that the observed effect on early development must be something specific, or a process which becomes limiting in this family, but is not as essential in *Solanaceae* or *Asteraceae*. The use of PGPR or mycorrhiza is very limited in *Brassicaceae* crops due to the high content in glucosinolates and sulforaphanes, which is very toxic for symbiotic microorganisms. So, the observed fungicidal effect of the yucca extracts described in this report should not be a problem when used in *Brassicaceae* crops, as symbiotic microorganisms are not routinely used. 

The difference between the two extracts’ used is the concentration of saponines, being higher in YUE 10 than YUE 8. We have seen that at the same concentration of extract YUE 10 is more effective than YUE 8 in sodium chloride in Arabidopsis (Figure 3a) and in mannitol in broccoli (Figure 3b). This suggests that saponin content may explain the biostimulant effect, at least in brassicaceae. The strongest effect was observed under NaCl and LiCl, indicating that at the molecular level the effect may be exerted at preventing the ionic stress (Figure 3a,b). The effect under mannitol stress conditions is much lower (Figure 3b,c) or even YUE 10 may have an inhibitory effect under mannitol stress in Arabidopsis (Figure 3a). On the other hand, saponins are toxic at high concentrations, and this may explain the fact that under lithium chloride conditions YUE 8 is more effective than YUE 10 (Figure 3a,b). The saponin content also explains the antifungal effect, as it was higher in YUE 10 than in YUE 8 at identical extract concentration (Figure 5). Another interesting outcome of the present study is that yucca extracts were conferring sodium chloride tolerance at the germination and early development level, but when we tested it in adult plants the effect was irrelevant. Plant physiology is very different at each developmental level, therefore is not so strange that a biostimulant may be useful at the early stage and irrelevant or even toxic at later stages of development. This observation supports the idea that every marketed biostimulant must be individually tested and at different level in order to grant its effectivity to farmers. In addition, given that yucca is a plant well-adapted to arid environments, and that the industrial cultivation is already set up in some countries (i.e., Mexico), the description of novel added-value products from *Yucca schidigera*, such as biostimulant or natural fungicides may be a great help to farmers working in arid environments.

## 4. Conclusions

In this report we have characterized and quantified two yucca extracts differing in their saponin content as biostimulants promoting germination and early vigor, mainly in *Brassicacea* plants. We have found that yucca extract increased germination under normal conditions, salt stress (lithium or sodium chloride) and water stress (mannitol) in Arabidopsis and also under stress conditions in broccoli. At later stages yucca extracts were able to increase the water content and the efficiency of photosystem II under lithium chloride or mannitol stress in Arabidopsis. We have also found that yucca extracts have a fungicide effect at a concentration above 8 mg/mL. Altogether our results indicate that yucca extracts may be a natural alternative to promote germination and early vigor, as well as a protection against fungi that may be used in conventional or organic farming practices. 

## 5. Materials and Methods

### 5.1. Yucca Plant Extracts

Two dry extracts of yucca plant (YUE 8 and YUE 10), provided by the Caldic Ibérica S.L.U company (Barcelona, Spain), were evaluated for their biostimulant effect on plant growth. Stock solutions were made from the dry product at a concentration of 10 mg/mL (*w*/*v*). Each stock solution was sterilized by tyndallization for three consecutive days before use.

### 5.2. Seed Germination, Plant Media and Growth Conditions

A detailed description for Arabidopsis can be found in [48] and broccoli in [49]. Seeds of *Arabidopsis thaliana* wild-type (ecotype Columbia-0), tomato (*Solanum lycopersicum* cv Micro-Tom), lettuce (*Lactuca sativa* L. cv. Romana) and Broccoli (*Brassica oleracea* var. Italica (provided by ‘Sakata Seed Iberica’)) were used to evaluate the germination-promoting effect by YUE 8 and YUE 10. Seeds were surface-sterilized with commercial bleach diluted 1:1 (*v*/*v*) for 15 min and rinsed with sterile water. The stratification was carried out for three days at 4 °C and subsequently, they were introduced into plates of Agar-Water medium containing 0.8% phytoagar. Standard petri dishes (15 × 100 mm) containing 20 mL of medium were used. Stress and/or Biostimulant were present in the phytoagar-water plates where seeds were deposited after stratification. Thirty seeds were placed in each plate with Agar-Water medium and were grown under long-day chamber conditions (16 h light/8 h dark, 23 °C, 130 μE m^−2^ s^−1^, 70% relative humidity). When indicated, the medium was supplemented with NaCl, LiCl or mannitol, as indicated in each case, and biostimulant products individually according to the assay. Expanded cotyledons data under different conditions were recovered per *visum* after 6 days. Each assay was performed in triplicate.

### 5.3. Establishment of the Toxic Dose of Yucca Extracts and the Optimal Concentration of the Abiotic Stressor

Seed germination and early vigor assays were performed to determine the toxic dose of yucca extracts under normal conditions and the possible biostimulant effect on seedling growth. From the stock solution, the following concentrations of yucca extract were tested: 20, 8, 1.6, 0.8 and 0.4 mg/mL. On the other hand, the optimal concentration of abiotic stressor was evaluated to observe the biostimulant effect of yucca extracts. For this, three concentrations of NaCl (80, 100 and 140 mM), LiCl (15, 20, 25 mM) and mannitol (240, 280 and 300 mM) were tested. All assays were carried out in Agar-Water media.

### 5.4. Treatment of Arabidopsis Seedling with Yucca Biostimulant under Saline and Osmotic Conditions

Wild-type *Arabidopsis thaliana* seeds (Columbia-0 ecotype) were superficially sterilized and stratified for three days at 4 °C. Seeds were germinated for 10 days in MS medium (Murashige and Skoog (MS) basal salt mixture (0.22%; Duchefa Biochemie B·V, Haarlem, The Netherlands), sucrose (1%), 2.6 mM MES (2-(N-morpholino) ethanesulfonic acid) buffer and 0.8% phytoagar, adjusted to pH 5.9 with potassium hydroxide). To measure the effect of yucca extracts on Arabidopsis growth to adulthood, seedlings were placed on previously hydrated 42-mm Jiffy-7 pellets. Irrigation was performed three times per week two with water and one with Hoagland complete solution, with 300 mL of water to the tray with the jiffies. Three days later, 1ml per pellet of the YUE 8 and YUE 10 extracts was applied at a concentration of 0.8 mg/mL (*w*/*v*). The application of stress by irrigation was carried out 10 days after transplanting the seedlings to the Jiffy pellets in the irrigation water with the indicated concentration of salt or mannitol until plants reached the silique stage. The stressors used were 140 mM NaCl and 24 mM LiCl (salt stress) and 280 mM mannitol (osmotic stress). Plants were incubated under long-day chamber conditions (16 h light/8 h dark, 23 °C, 130 μE m^−2^ s^−1^, 70% relative humidity). The fresh and dry weight, the percentage of plants with flowers and fruits, and the water content in the adult plants were recorded in pants in the silique stage. The adult plants were oven dried at 70 °C until a constant weight was achieved. The weight was measured using an analytical balance. The percentage of water content (WC) was calculated using the following formula: ((FW-DW)/FW), where FW is the fresh weight and DW is the dry weight and is expressed as %H_2_O. Chlorophyll a yield indexes were measured with the HandyPEA fluorimeter (Hansatech, Pentney, England).

### 5.5. Antibiogram

The antifungal effect of yucca extracts was evaluated. Media for yeast growth and cultivation were prepared as described in [50]. For this, a pre-culture of the baker’s yeast strain BY4741 was carried out in Yeast extract Peptone Dextrose (YPD) broth (1% yeast extract, 2% bacteriological peptone and 2% glucose). The culture was incubated at 28 °C, 200 rpm for two days. Subsequently, YPD (1% yeast extract, 2% bacteriological peptone, 2% glucose, and 2% agar in distilled water) plates were inoculated with 50 µL of a 1:5 dilution of the yeast saturated culture. The inoculum was spread homogeneously over the agar surface. Finally, Whatman disks impregnated with different concentrations (40, 20, 8, 1.6, 0.8 and 0.6 mg/mL) of YUE 8 and YUE 10 were applied on the agar surface. Then the soft agar inoculated with the yeast was poured on the plate The plates were incubated for two days at 28 °C and the diameter of inhibition halos were measured using ImageJ software (National Institute of Mental Health, USA).

### 5.6. Statistical Analysis

Student’s test was performed using R software. These tests were calculated concerning the increase or decrease in plant biomass compared to the control. The means are considered to be significantly different at *p* < 0.05.

## Data Availability

All the data presented in this study has been used in the figures.
